# CDK 4/6 Inhibition Overcomes Acquired and Inherent Resistance to PI3K*α* Inhibition in Pre-Clinical Models of Head and Neck Squamous Cell Carcinoma

**DOI:** 10.3390/jcm9103214

**Published:** 2020-10-07

**Authors:** Eric Remer, Mai Badarni, Elad Hikri, Avraham Dayan, Lirit Levi, Aron Popovtzer, Muhammed Iraqi, Angel Porgador, Ben-Zion Joshua, Gideon Bachar, Moshe Elkabets, Maurizio Scaltriti, Aviram Mizrachi

**Affiliations:** 1 Department of Otolaryngology-Head and Neck Surgery, Meir Medical Center, Kfar Saba 4428164, Israel; ericremer@gmail.com; 2 Sackler Faculty of Medicine, Tel Aviv University, Tel Aviv 6997801, Israel; eladhi@clalit.org.il (E.H.); avrahamda1@clalit.org.il (A.D.); liritco@clalit.org.il (L.L.); aronp@clalit.org.il (A.P.); GideonB@clalit.org.il (G.B.); 3 The Shraga Segal Department of Microbiology, Immunology and Genetics, Faculty of Health Sciences, Ben-Gurion University of the Negev, Beer-Sheva 84105, Israel; maibdarny@gmail.com (M.B.); iraqi@post.bgu.ac.il (M.I.); angel@bgu.ac.il (A.P.); moshe.elkabets@gmail.com (M.E.); 4 Department of Otolaryngology-Head and Neck Surgery, Rabin Medical Center, Petah Tikva 49100, Israel; 5 Davidoff Cancer Center, Rabin Medical Center, Petah Tikva 49100, Israel; 6 Department of Otolaryngology-Head and Neck Surgery, Soroka University Medical Center, Beer-Sheva 84011, Israel; benzionj@gmail.com; 7 Human Oncology & Pathogenesis Program, Memorial Sloan-Kettering Cancer Center, New York, NY 10021, USA; scaltrim@mskcc.org

**Keywords:** head and neck squamous cell carcinoma, human papillomavirus, *PIK3CA*, PI3K, CDK 4/6, mTOR

## Abstract

Activating alterations in *PIK3CA*, the gene coding for the catalytic subunit of phosphoinositide-3-kinase (PI3K), are prevalent in head and neck squamous cell carcinoma (HNSCC) and thought to be one of the main drivers of these tumors. However, early clinical trials on PI3K inhibitors (PI3Ki) have been disappointing due to the limited durability of the activity of these drugs. To investigate the resistance mechanisms to PI3Ki and attempt to overcome them, we conducted a molecular-based study using both HNSCC cell lines and patient-derived xenografts (PDXs). We sought to simulate and dissect the molecular pathways that come into play in *PIK3CA*-altered HNSCC treated with isoform-specific PI3Ki (BYL719, GDC0032). In vitro assays of cell viability and protein expression indicate that activation of the mTOR and cyclin D1 pathways is associated with resistance to PI3Ki. Specifically, in BYL719-resistant cells, BYL719 treatment did not induce pS6 and pRB inhibition as detected in BYL719-sensitive cells. By combining PI3Ki with either mammalian target of rapamycin complex 1 (mTORC1) or cyclin D1 kinase (CDK) 4/6 specific inhibitors (RAD001 and abemaciclib, respectively), we were able to overcome the acquired resistance. Furthermore, we found that PI3Ki and CDK 4/6 inhibitors have a synergistic anti-tumor effect when combined in human papillomavirus (HPV)-negative/*PIK3CA*-WT tumors. These findings provide a rationale for combining PI3Ki and CDK 4/6 inhibitors to enhance anti-tumor efficacy in HNSCC patients.

## 1. Introduction

Head and neck squamous cell carcinoma (HNSCC) is the sixth most common malignancy worldwide, affecting ~600,000 patients per year [1,2]. The curative treatment modalities for most HNSCCs consist of surgery and/or radiation therapy (RT), while chemotherapy may be added in advanced-stage cancers and recurrent disease [3]. Despite advances in basic and clinical research, standard treatments for HNSCC including surgery, radiation, and chemotherapy have not significantly altered patients’ overall survival, which remains stable at ~50% in recent decades [4]. Genetic alterations associated with the deregulation of the cell-cycle-regulatory machinery are detected in nearly all cases of HNSCCs [5,6]. An example of these findings is the detection of aberrations within *TP53, CDKN2A, PIK3CA, NOTCH*, and *HRAS* oncogenes in a recent genetic analysis of HNSCC [5].

The phosphoinositide 3-kinase (PI3K) is essential for the regulation of cell cycle, proliferation, survival, and metabolism. This pathway integrates many extracellular stimuli and triggers the phosphorylation of key downstream effectors such as AKT and the mammalian target of rapamycin complex 1 and 2 (mTORC1 and 2) [7,8]. Activation of PI3K results in increased phosphatidylinositol-(3,4,5)-triphosphate (PIP_3_) at the plasma membrane, promoting the recruitment of the pleckstrin homology domain-containing proteins Pyruvate dehydrogenase kinase (PDK) and AKT, [9,10] where the constitutively active kinase PDK1 phosphorylates AKT at the activation loop (T308) [11] and mTORC2 in the hydrophobic motif (S473) [12]. Once active, AKT phosphorylates a variety of anti-apoptotic and cell-cycle-related proteins as well as transcription factors **[13]**. In addition, AKT activates downstream mTORC1 through the phosphorylation of the negative regulators TSC2 and PRAS40 [14,15,16,17].

Activating mutations in *PIK3CA* are prominent in HNSCC, occurring in 34–56% of cases [5]. *PIK3C*A encodes the α isoform of the p110 catalytic subunit of PI3K, and mutations or copy number amplifications result in the hyperactivation of the PI3K/AKT/mTOR pathway [7]. These alterations are also common in breast cancer, [18] prostate cancer [19], and colorectal cancer [20]. These mutations are validated therapeutic targets, and selection for *PIK3CA* alterations leads to superior anti-tumor activity of PI3K inhibitors (PI3Ki), resulting in tumor stabilization and, in some cases, regression [21].

**Figure 1:**
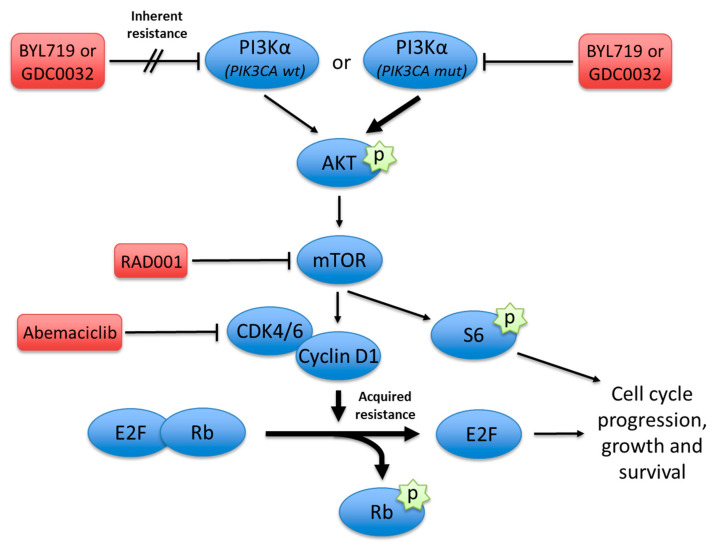
Graphical abstract depicting the mechanisms of inherent and acquired resistance to PI3K inhibition at different vertical nodes along the PI3K/Akt/mTOR pathway. Activating mutations and copy number amplifications in *PIK3CA* result in marked upregulation of PI3Kα signaling. Acquired resistance develops via activation of the CDK 4/6–cyclin D1 complex that may be blocked simultaneously to overcome this resistance. Alternatively, mTOR inhibition may synergize downstream with PI3Kα inhibition. Conversely, *PIK3CA* wild-type tumors are independent of PI3Kα activity, which makes them inherently resistant to PI3Kα inhibition. Hence, combining additional inhibition along the pathway may overcome this resistance and improve the efficacy of PI3K inhibitors.

Recent pre-clinical reports have suggested that acquired resistance to PI3K inhibition is a result of the activation of alternative pathways such as upregulation of receptor tyrosine kinases that activate the mTORC1 [22] and the Cyclin D1 kinase (CDK) complex (Figure 1) [23]. This study aims to investigate the role of the CDK/Rb axis in conferring resistance to PI3Ki in HNSCC. Moreover, we investigated therapy combinations with CDK 4/6 inhibitors to overcome PI3Ki resistance in vitro and in pre-clinical models.

## 2. Materials and Methods

### 2.1. Experimental Design and Setting

All in vitro and in vivo experiments were performed at the Center for Translational Research in Head and Neck Cancer, Felsenstein Medical Research Center, Rabin Medical Center.

The experimental drugs used in this study are the PI3K inhibitors, BYL719 (alpelisib) and GDC0032 (taselisib); the mTOR inhibitor, RAD001 (everolimus); and the CDK 4/6 inhibitor, abemaciclib (LY2835219).

All experimental drugs used in this study were provided by Dr. Maurizio Scaltriti from the Human Oncology and Pathogenesis Program at Memorial Sloan-Kettering Cancer Center, New York, NY, USA.

### 2.2. Cell Cultures

All cell lines were obtained from the ATCC. Cell lines were maintained in humidified incubators at 37 °C in the appropriate cell culture media supplemented with 10% heat-inactivated fetal calf serum, 2 mM L-glutamine, penicillin (20 U/mL), and streptomycin (20 mg/mL).

### 2.3. Determination of PIK3CA Mutation and Copy Number Status

*PIK3CA* mutation and amplification status information for cell lines was obtained from the Cancer Cell Line Encyclopedia (www.broadinstitute.org/ccle). Patient-derived xenografts were sequenced using the MSK-IMPACT™ next-generation sequencing platform. Amplification was defined as greater than or equal to four copies of the *PIK3CA* gene.

### 2.4. Western Blot

Cells lysates were subjected to sodium dodecyl sulfate–polyacrylamide gel electrophoresis (SDS-PAGE), transferred to a nitrocellulose membrane and immunoblotted with the appropriate primary antibodies as anti-phospho-activated and general PI3K, AKT, m-TOR, and other downstream signaling candidates.

### 2.5. XTT Cell Proliferation Assay

Cells were seeded at 5 × 10^3^ cells/well into 96-well plates to a final volume of 100 μL and allowed to grow for the required periods of time with the appropriate treatments. Then, the activated XTT reaction solution was added to each well for an additional 2–4 h incubation at 37 °C. Finally, the reaction was measured at a wavelength of 450 nm against a background control by a microplate reader.

### 2.6. Establishment of Tumor Xenografts

Patient-derived xenografts (PDXs) were established from HNSCC patients treated at the Ear, Nose, and Throat Unit, Soroka Medical Center (SE20 and SE103) or Memorial Sloan-Kettering Cancer Center (H22). All patients harbored SCC of the larynx and signed informed consent forms. All PDXs were first transplanted s.c. into the flanks of 6-week-old NOD.Cg-Prkdc Il2rg/SzJ (NSG) or nude mice. Upon successful tumor engraftment, tumors were expanded and retransplanted into immunodeficient mice for drug efficacy experiments. About 2 weeks after the second transplantation, the mice were randomized into 4 groups of 6–8 mice per group. NSG mice were purchased from The Jackson Laboratory.

### 2.7. In Vivo Studies

All animal experiments were carried out in compliance with the Israel council on animal care regulations and were approved by the animal care committee of Rabin Medical Center. HNSCC cell lines (2 × 10^6^) or PDXs of a tumor obtained from a treatment-naïve patient with larynx squamous cell carcinoma were implanted subcutaneously into the back-flank area of nude mice. Tumor volume was measured using a digital caliper twice weekly during the entire treatments. Tumor growth curves were calculated using the formula (length × width^2^)/2.

### 2.8. In Vitro Synergistic Anti-Tumor Effect

Cells were seeded in 96-well plates (3000 cells per well), treated with increasing concentrations of the indicated drugs (0–10 μM BYL719, 0–2 μM GDC0032, and 0–10 μM abemaciclib), and allowed to proliferate for 4 days. Cells were then fixed and stained with crystal violet (1 g/L) for 30 min at room temperature. Following rinsing, the bound crystal violet was dissolved out with 10% acetic acid, and absorbance was measured at 570 nm (BioTek Epoch spectrophotometer). The proliferation of the cells in the different treatment groups was presented as a percentage of control (DMSO-treated) cells, and the percent of growth inhibition was calculated. Zero interaction potency (ZIP) Synergy scores were calculated using SynergyFinder 2.0 (https://doi.org/10.1093/nar/gkaa216).

### 2.9. Tumor Ex Vivo Analysis (TEVA)

Tumor ex vivo analysis (TEVA) was performed when the volume of PDXs (SE20 and SE103) reached ~500 mm^3^. The PDXs were excised out aseptically from mice and cut into 2 × 2 × 2 mm^3^ tissue explants. The 2 × 2 × 2 mm^3^ explants were then incubated with different therapeutic drugs for 24 h in 48 well tissue culture plates at 37 °C, 95% relative humidity, and 5% CO_2_ incubator at sterile conditions with suitable control [24]. The 2 × 2 × 2 mm^3^ explants incubated only in culture media without any drug served as control. Dulbecco’s Modified Eagle’s Medium (DMEM, Gibco) containing 20% FBS (Gibco), 1 mM sodium pyruvate (Biological Industries), 2 mM L-glutamine (Biological Industries), 1% penicillin–streptomycin–amphotericin (Biological Industries), 0.1 mM MEM non-essential amino acids (Biological Industries), 10 mM HEPES (Biological Industries), 1% BIO-MYC (Biological Industries) and 50 µg/mL gentamycin (Gibco) were used as culture media. Drug concentrations of BYL719 (2 µM), GDC0032 (500 nM), abemaciclib (1 µM) were used for the study as therapeutic agents. For calculating the TEVA score, KI67, TUNEL, and pS6 was assessed as previously described [24].

The antibodies for the TEVA were KI67 (1:250, Vector laboratories, cat no-VP K451), TUNEL assay was done according to manufacturer protocol (TREVIGEN, cat no-4815-30-K), and pS6 cell signaling protein (Ser240/244) (D68F8) XP^®^ rabbit mAb #5364.

### 2.10. Statistical Analysis

SPSS version 23 software (Armonk, NY, USA) and Graphpad Prism version 6.0 (La Jolla, CA, USA) were used to analyze and plot the data. Data were presented as mean ± standard error (SE). Differences between two groups were analyzed using the independent t-test, whereas the analysis of variance (ANOVA) test was used to compare multiple groups. The Bonferroni method of correction was applied for multiple comparisons.

## 3. Results

### 3.1. Sustained Rb Phosphorylation Is Associated with Resistance to PI3K Inhibition

**Figure 2:**
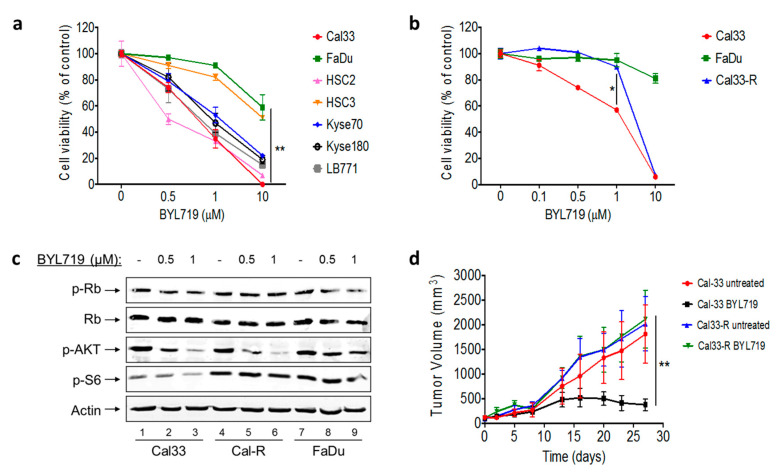
Mechanism of acquired resistance to phosphoionositide 3 kinase (PI3K) inhibition (**a**) Cell viability curves of different head and neck squamous cell carcinoma (HNSCC) and esophageal SCC cell lines (*PIK3CA* mutated, amplified, and wild type) treated with BYL719. (**b**) Cell viability curves of Cal33, FaDu, and Cal33-R cell lines treated with BYL719. (**c**) Signaling pathway protein expression in Cal33, Cal33-R, and FaDu cell lines treated with BYL719. (**d**) Tumor growth curves of Cal33 and Cal33-R cell line xenografts treated for 27 days with daily oral administration of either 50 mg/kg BYL719 or vehicle (*n* = 8). (* *p* < 0.05, ** *p* < 0.01).

We initially tested seven different HNSCC and esophageal SCC cell lines in vitro for sensitivity to the alpha-specific PI3Ki BYL719 (Figure 2a). As predicted, enhanced sensitivity was noted in cell lines with *PIK3CA* mutations or amplifications. In order to explore the durability of response to PI3K inhibition, we selected the Cal33 cell line, which harbors an activating *PIK3CA* mutation (H1047R). By prolonged exposure to low doses of BYL719, we created a BYL719-resistant cell line (Cal33-R). We then compared cell viability between Cal33, Cal33-R, and FaDu, a *PIK3CA*-amplified cell line, when exposed to increasing concentrations of BYL719. These experiments confirmed that Cal33-R is resistant to BYL719, with a drug sensitivity comparable to *PIK3CA*-WT cells (Figure 2b).

Next, we studied the activity of BYL719 in perturbing the PI3K signaling pathway and the phosphorylation of Rb. While the inhibition of phosphorylation of Akt was similar in both Cal33 and Cal33-R treated with BYL719, phosphorylation of S6 and Rb, the main readout of mTORC1 and CDK inhibition, was sustained in the presence of BYL719 in Cal33-R (Figure 2c).

We then tested whether the resistance phenotype of Cal33-R was maintained in vivo. Tumors from Cal33 and Cal33-R cells were engrafted in mice and treated with either 50 mg/kg/day BYL719 or vehicle for 27 days. Similar to the in vitro results, the Cal33-R xenografts were resistant to PI3Ki, while Cal33 tumors showed durable growth inhibition (Figure 2d).

### 3.2. Blocking mTORC1 or CDK 4/6 Overcomes Resistance to PI3Ki

**Figure 3:**
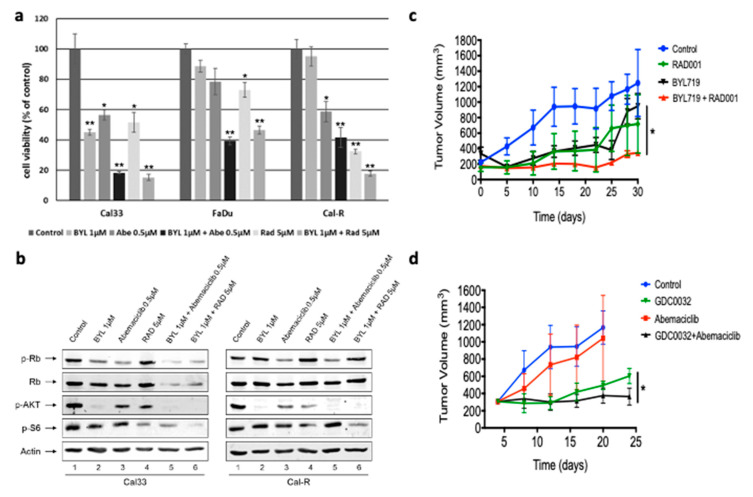
Overcoming resistance to PI3Ki by blocking mammalian target of rapamycin complex 1 (mTORC1) or cyclin D1 kinase (CDK) 4/6. (**a**) Cell viability curves of Cal33, FaDu, and Cal33-R cell lines, treated with either BYL719, abemaciclib, or RAD001 monotherapy or combination. (**b**) Signaling pathway protein expression in Cal33 and Cal33-R cell lines, treated with either BYL719, RAD001, and abemaciclib monotherapy or combination therapy. (**c**) Tumor growth curves of H22 patient-derived xenografts treated for 30 days with daily oral administration of either 50 mg/kg BYL719, 0.5 mg/kg RAD001, or a combination of both drugs (*n* = 10). (**d**) Tumor growth curves of H22 patient-derived xenografts treated for 25 days with daily oral administration of either 5 mg/kg GDC0032, 50 mg/kg abemaciclib, or a combination of both drugs (*n* = 10). (* *p* < 0.05, ** *p* < 0.01).

In an attempt to overcome the acquired resistance to PI3Ki, we sought to exploit the resistance mechanism demonstrated earlier by using the CDK 4/6 inhibitor, abemaciclib, and an mTORC1 inhibitor, RAD001. First, we conducted cell viability assays in Cal33, Cal33-R, and FaDu cell-lines in vitro using a combination of BYL719 with either abemaciclib or RAD001 (Figure 3a). Both combinations significantly decreased cell viability in Cal33-R compared to treatment with BYL719 only.

When looking at how these drug combinations affect the signaling pathway, we observed that, when BYL719 is combined with abemaciclib, the phosphorylation of Rb is attenuated without affecting the phosphorylation of S6 in Cal33-R, whereas BYL719 combined with RAD001 resulted in decreased phosphorylation of S6 without affecting Rb phosphorylation (Figure 3b).

We then sought to correlate these findings in vivo using a patient-derived xenograft (PDX) model of *PIK3CA*-mutated HNSCC, named H22, by testing the efficacy of therapy combinations of BYL719 or GDC0032 with RAD001 or abemaciclib, respectively (Figure 3c,d). Both experiments demonstrated tumor progression in mice treated with BYL719, GDC0032, RAD001, and abemaciclib. However, combining PI3Ki with either mTORC1 or CDK 4/6 inhibitors achieved superior tumor growth control.

### 3.3. Combination Treatment with PI3K and CDK 4/6 Inhibitors Display a Synergistic Effect in PIK3CA-WT Tumors

**Figure 4:**
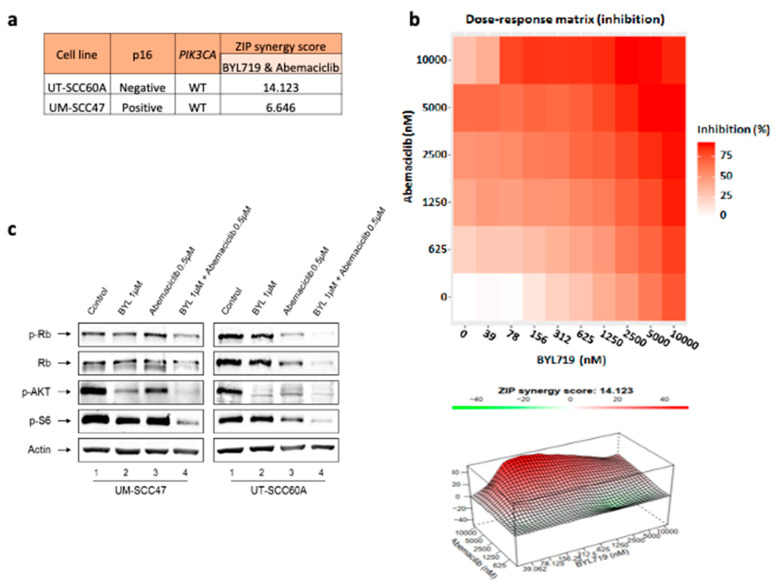
Combination treatment with PI3K and CDK 4/6 inhibitors display a synergistic effect in *PIK3CA*-WT tumors. (**a**) Average ZIP synergy scores, calculated by SynergyFinder 2.0, of UT-SCC60A and UM-SCC47 following treatment with a combination of abemaciclib and BYL719. (**b**) Dose–response matrix and ZIP synergy score surface plots of UT-SCC60A following treatment with a combination of BYL719 and abemaciclib. (**c**) Signaling pathway protein expression in UM-SCC47 and UT-SCC60A following treatment with either BYL719 or abemaciclib monotherapy or combination therapy.

To further understand the dual inhibitory effect of PI3K and CDK 4/6, we tested whether abemaciclib in combination with BYL719 is additive or synergistic in two *PIK3CA-*WT cell lines, one p16- UT-SCC60A and one p16+ UM-SCC-47. Specifically, we performed a synergy test using SynergyFinder 2.0 with the ZIP model [25], which enables scouring of all combined concentrations simultaneously, which allows us to look at the overall landscape and gain a complete understanding of the interaction between the drugs. The average ZIP synergy score showed that the combination of BYL719 and abemaciclib has a synergistic effect in UT-SCC60A, as the synergy score was 14.123. The synergy score in UM-SCC-47 was lower, 6.646 (Figure 4a). The synergy indicated by 3D surface plots showing regions of average ZIP synergy scores alongside with dose–response matrix (Figure 4b).

Aiming to understand the molecular mechanism behind the observed synergistic phenotype, we performed WB analysis following dual exposure to BYL719 and abemaciclib. We saw enhanced inhibition of the PI3K/Akt/mTOR and Rb pathways, as indicated by pS6, pAKT, and pRB following administration of the drug combinations in p16- UT-SCC60A and to a lesser extent in p16+ UM-SCC47 (Figure 4c).

### 3.4. Combination of PI3K and CDK 4/6 Inhibitors Display Synergistic Effect in a PDX Ex Vivo Model

**Figure 5:**
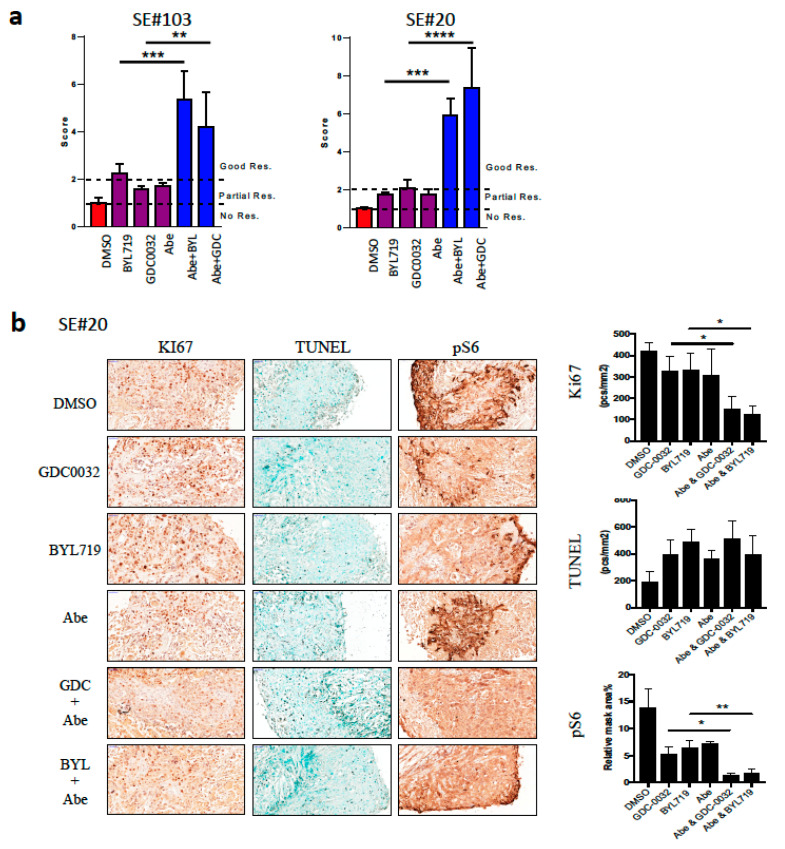
Combination of PI3K and CDK 4/6 inhibitors display synergistic effect in a patient-derived xenograft (PDX) tumor ex-vivo analysis (TEVA). (**a**) TEVA score analysis of SE#20 and SE#103 at 24 h following exposure to either BYL719, GDC0032, and abemaciclib monotherapy or combination therapy. (**b**) Representative images of immunohistochemistry staining for KI67, TUNEL, and pS6 in SE#20. (* *p* < 0.05, ** *p* < 0.01, *** *p* < 0.001, **** *p* < 0.0001).

To reinforce our findings in additional pre-clinical models, we performed ex vivo efficacy studies combining BYL719 or GDC0032 with abemaciclib using the tumor ex-vivo analysis (TEVA) [24]. Briefly, fragments of PDXs, exposed to drugs for 24 h, and tumor cells viability and signaling pathway activation are measured by KI67, TUNEL, and pS6. The tumor viability and pathway activation was calculated for the ex vivo score as previously defined [24]. We used two human papilloma virus negative (HPV−)/*PIK3CA-*WT PDXs, #20 and #103, to explore how the combination of GDC0032 or BYL719 with abemaciclib will affect pathway inhibition and tumor viability. Specifically, we performed IHC staining for KI67, TUNEL, and phosphorylation of S6. TEVA score analysis showed that the combination has a superior anti-tumor activity compared to any single agent in the two mice, with an ex vivo score above 2 (Figure 5a). Representative images of staining and analysis of PDX #20 are shown here (Figure 5b).

## 4. Discussion

Previous studies in HNSCC as well as in other cancers that harbor activating mutations in *PIK3CA* have found these genetic alterations only targetable to a limited extent [26,27,28]. These data raised the concern that the complexity of the PI3K/Akt/mTOR pathway does not allow durable inhibition with monotherapy of PI3Ki [29]. In the present study, we show that blocking CDK 4/6 may overcome the acquired and inherent resistance to PI3Ki, and in pre-clinical HNSCC models, dual treatment with PI3Ki and CDK 4/6i induced durable clinical responses.

The rationale for combining pathways inhibitors to prevent PI3Ki resistance has been shown to achieve extended response in pre-clinical HNSCC models. For example, by simultaneously blocking mTORC2 [30,31], human epidermal growth factor receptor 3 (HER3) [32,33], Epidermal growth factor receptor (EGFR) [34,35], Jun N-terminal kinases (JNK) [36], Extracellular signal-regulated kinases (ERK) [37], and AXL [22]. Previously, Vora et al. [23] and others [26] described in both pre-clinical breast cancer models and in patients, that resistance to BYL719 is mediated by sustained activation of mTORC1 that regulates the CDK 4/6–Rb axis. This mTORC1 activation in an AKT-independent manner is regulated by Serine/threonine-protein kinase 1 SGK1 in breast cancer, [38] and by upregulation of AXL that activates mTORC1 via Protein kinase C (PKC ) in HNSCC [22].

In the present study, we sought to investigate whether the CDK 4/6–Rb axis itself plays a role in the mechanism of resistance to PI3Ki in HNSCC. First, we looked at the levels of pRB following exposure to PI3Ki in sensitive as well as acquired-resistant models (Cal33 and Cal33-R) and found that pRB is elevated in Cal33-R. Subsequently, we tested whether blocking CDK 4/6 can enhance PI3Ki efficacy in vitro and in vivo and demonstrated that the two drugs work in a synergistic fashion in vitro, while in vivo and ex vivo, the combination was more potent than any single agent. Second, we know that the inactivation of the cyclin D1 complex plays a crucial role in the carcinogenesis of HNSCC. Interestingly, we found that resistance to PI3Ki has many shapes and forms, either acquired following prolonged exposure or inherent in *PIK3CA*-WT tumors. Moreover, HPV-related tumors have high levels of p16 and low levels of pRB, which explains why CDK 4/6 inhibitors are less effective in these virus-driven cancers [39]. While HPV-tumors are driven by various genetic alterations, the most prominent occurring in the *TP53* gene, in HPV+ tumors, Rb is inactivated by the viral oncoprotein E7, hence these tumors are less sensitive to inhibition of CDK 4/6. Conversely, in *PIK3CA*WT tumors that are inherently resistant to PI3Ki and are also HPV−, combining PI3Ki with CDK 4/6 inhibitors may overcome this resistance. This was well demonstrated by significant in vivo and ex vivo anti-tumor effect in *PIK3CA*-mutated PDXs, which acquired resistance over time upon exposure to PI3Ki as well as *PK3CA*-WT PDXs treated with combination therapy.

Multiple reports have highlighted the need to block CDK 4/6 in order to enhance therapeutic efficacy. Similarly, investigators found that combination treatment with CDK 4/6 and mTOR inhibitors has a potential therapeutic effect in HNSCC. This synergistic effect is the result of a downstream blockade of E2F by both mTOR inhibitors, and since the CDK 4/6 pathway regulates cellular proliferation by controlling the G1 to S cell cycle checkpoint [40,41,42,43]. Moreover, CDK 4/6 has been shown to be a potential radiosensitizer that can enhance the cytotoxic effects of radiation therapy [44]. Furthermore, in HNSCC, the Rb pathway is frequently altered through amplification of Cyclin D1 or deletion of *CDKN2A*, and thus promoting proliferation.

That being said, it is still unclear whether HNSCC patients will benefit from CDK 4/6 inhibitor monotherapy, but we anticipate that p16 loss and cyclin D1 amplification might serve as biomarkers for selecting patients for such treatment [43].

Finally, in HPV− patients who have exhausted platinum- and/or cetuximab-based regimens, combining PI3K and CDK 4/6 inhibitors might be the last resort even when they do not harbor any known activating mutations [45].

In conclusion, the present study demonstrates the importance of blocking CDK 4/6 in order to enhance the efficacy of PI3Ki in HNSCC, specifically HPV− tumors. Given the prevalence of *PIK3CA* alterations in HNSCC and its dominant driving properties, understanding mechanisms of response and resistance to PI3K therapies is paramount for the future of precision medicine in these patients. We believe that these findings lay the ground for the rationale of combination strategies, which are based on a molecular landscape and may be readily translated to the clinic.
